# Enhanced Time‐Locked Decoding for Spoken Words but Not Environmental Sounds in Natural‐Like Auditory Conditions

**DOI:** 10.1111/ejn.70598

**Published:** 2026-07-03

**Authors:** Jesper Edström, Anni Nora, Oona Rinkinen, Riitta Salmelin, Hanna Renvall

**Affiliations:** ^1^ Department of Neuroscience and Biomedical Engineering Aalto University Espoo Finland; ^2^ BioMag Laboratory, HUS Diagnostic Center Aalto University, University of Helsinki, and Helsinki University Hospital Helsinki Finland

**Keywords:** auditory attention, magnetoencephalography, speech processing, stimulus reconstruction

## Abstract

Humans are especially sensitive to speech sounds even in complex acoustic environments, but it remains unclear whether speech is tracked differently from other meaningful sounds under such conditions. Magnetoencephalography recordings combined with machine learning have revealed that dynamic time‐locking of cortical activation to unfolding speech is crucial for encoding its acoustic–phonetic features. Here we investigated whether a similar mechanism for speech encoding operates during concurrent processing of speech and nonspeech sounds. Twenty participants listened to superimposed spoken words and nonspeech environmental sounds while attending to one stream at a time. Using a time‐locked decoding model, we reconstructed time‐varying acoustic characteristics of attended and ignored sounds from neural responses in each hemisphere. In the left hemisphere, amplitude envelopes of attended spoken words were decoded significantly better than those of attended environmental sounds at 120‐ to 200‐ms latency between the sound and the cortical activation. No such difference between sound types emerged in the right hemisphere. Furthermore, attention significantly enhanced the decoding of speech sounds in the left hemisphere, suggesting that the observed effects reflect both bottom‐up and top‐down driven processes. These findings imply that particularly the left hemisphere processes speech sounds in a special, time‐locked manner even under adverse auditory conditions. This mechanism may share its neural underpinnings with the previously reported time‐locked tracking of speech amplitude envelope by cortical oscillatory activation or by evoked responses to acoustic edges, supporting efficient extraction of speech features in natural‐like listening conditions.

## Graphical abstract

We examined cortical tracking of speech under naturalistic auditory conditions by recording MEG responses while participants listened to superimposed spoken words and environmental sounds. Decoding sound envelopes and spectrograms from the MEG responses revealed a more accurate cortical tracking of speech compared to other natural sounds in the left hemisphere.

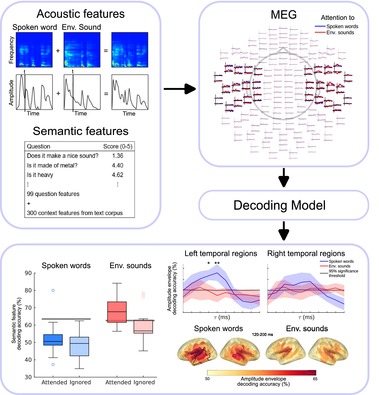

**Abbreviations**
ANOVA: analysis of variance
BEM: boundary element method
dSPM: dynamical statistical parametric mapping
ECG: electrocardiogram
EEG: electroencephalography
EOG: electrooculogram
FFT: fast Fourier transform
HPI: head position indicator
ICA: independent component analysis
MAP: maximum‐a posteriori
MEG: Magnetoencephalography
MRI: magnetic resonance image
RMS: root mean square
SD: standard deviation
SNR: signal‐to‐noise ratio
TRF: temporal response function
tSSS: spatiotemporal signal space separation

## 1 Introduction

Humans have a remarkable ability to understand speech even in complex listening conditions comprising background noise or multiple talkers. Under such conditions, speech perception depends on accurate segmenting and grouping of various temporal and spectral features into sound streams corresponding to the different sound sources (Bregman 1990), and likely relies on the interaction between bottom‐up stimulus‐driven processing and top‐down selective attention (Alain et al. 2001; Pichora‐Fuller et al. 1995; Zekveld et al. 2006). Neural oscillations in the delta (1–4 Hz) and theta (4–8 Hz) frequency bands synchronize to the amplitude fluctuations in the speech signal (Ahissar et al. 2001; Ding and Simon 2014; Luo and Poeppel 2007; Obleser and Kayser 2019), which is thought to facilitate the prediction of upcoming information, and has been linked to both repetitive evoked responses to acoustic edges in speech (Doelling et al. 2014; Oganian et al. 2023) and continuous entrainment of endogenous neural oscillations to the speech envelope (Giraud and Poeppel 2012; Schroeder and Lakatos 2009). Cortical tracking of speech depends on speech intelligibility (Ershaid et al. 2024; Luo and Poeppel 2007; Peelle et al. 2013), and is unparalleled in temporal accuracy compared to the tracking of other meaningful sounds such as music, animal sounds, and even nonverbal human sounds with similar temporal and spectral characteristics as speech (Nora et al. 2020; Zuk et al. 2021), suggesting that the brain tracks speech in a special manner.

Selective attention enhances the neural representation of attended speech while suppressing the representation of ignored sounds (Ding and Simon 2012a; Horton et al. 2013; Kerlin et al. 2010; Khalighinejad et al. 2019; Kong et al. 2014; Mesgarani and Chang 2012; Zion Golumbic et al. 2013). Consistently, the amplitude envelope of attended speech can be more accurately reconstructed from electroencephalography (EEG; Fuglsang et al. 2017; Mirkovic et al. 2015; O'Sullivan et al. 2015) and magnetoencephalography (MEG; Ding and Simon 2012b) recordings compared to the envelope of ignored speech. The observed cortical tracking of attended speech appears to be robust even in naturalistic listening conditions containing background noise (Desai et al. 2021; Wang et al. 2020) or reverberation (Fuglsang et al. 2017).

In this work, using a machine learning approach, we investigate whether spoken words are more closely tracked by neural responses as compared to simultaneously presented environmental sounds, and how possible differences in cortical tracking relate to selective attention. Prior work shows distinct cortical engagement when attending to speech versus other meaningful sounds in complex scenes (Renvall et al. 2021), yet it remains unclear whether these sound classes share or rely on separable cortical mechanisms when presented concurrently under comparable task and semantic processing demands. Addressing this question is directly relevant for designing auditory rehabilitation protocols that selectively target impaired processing streams, for example, in poststroke aphasia. Furthermore, it allows us to address whether the time‐locked cortical tracking of speech versus other meaningful sounds is mainly modulated by selective attention to the sounds or rather relates to acoustic characteristics of the stimuli.

We examined the time‐locked processing of speech and environmental sounds in complex listening conditions using MEG and a stimulus reconstruction model that assumes cortical responses to accurately track the unfolding acoustic sound features in time. The participants were presented with superimposed spoken words and environmental sounds, while they selectively attended to either the speech or environmental sound excerpts. This design enabled a direct within‐trial comparison of speech and environmental sound decoding which is not possible when the stimuli are presented in isolation. The semantic contents of the single spoken words and environmental sounds were matched to align semantic processing requirements, while manipulating the targeting of attention to the sounds. In this way, top‐down influences can be assumed to be related to attentional modulation. The models were trained to reconstruct time‐varying acoustic features, here the amplitude envelope and spectrogram, of the sound excerpts based on the left‐ and right‐hemispheric MEG responses. Both attended and ignored sound features were separately reconstructed to assess the influence of top‐down versus bottom‐up processing mechanisms. In parallel, we evaluated the reconstruction of the semantic features of the sounds using time‐independent models: measures of semantic processing were considered to provide control over possible differences in the attentional allocation between the sound excerpts. We expected the acoustic features of spoken words to be better decoded from the MEG responses compared to the environmental sounds, similarly to our previous study using isolated sounds (Nora et al. 2020), and that selective attention would preferentially enhance speech tracking relative to environmental sound tracking.

## 2 Materials and Methods

### 2.1 Participants

Twenty Finnish‐speaking right‐handed participants (12 women, eight men) aged 22–48 years (mean = 35, SD = 10) participated in the study. All participants reported normal hearing and no history of neurological disorders. The study had obtained a prior ethical statement from the Aalto University Research Ethics Committee and the participants gave their written consent before the experiment. The sample size (*N* = 20) was comparable to the corresponding MEG studies using speech and other natural sounds (see, e.g., Nora et al. 2020; Renvall et al. 2021). We also estimated the effect size r to be large (> 0.7–0.8) based on the *Z*‐values obtained in Nora et al. (2020), where a similar convolution model was applied to MEG responses to isolated speech and environmental stimuli and compared to non–time‐sensitive models on the same stimuli.

### 2.2 Stimuli

The stimuli consisted of 44 superimposed spoken words and environmental sounds that were equalized for their root mean square (RMS) intensity before superimposition, resulting in 0 dB signal‐to‐noise ratio (SNR) of the individual sounds. The stimuli were obtained from Nora et al. (2020) where they were used in isolation. The 44 spoken words were common Finnish nouns (e.g., “chicken,” “car,” and “guitar”) representing six different semantic categories (animals, human nonlinguistic sounds, tools, musical instruments, vehicles, and miscellaneous). The spoken words consisted of two to five syllables, and their mean duration was 810 ms (SD = 170 ms). The words were uttered by eight different speakers (four males, four females; two children) to eliminate the influence of speaker‐specific acoustic features on the results. The uniqueness point (i.e., the point at which the word diverges from all the other words) was on average 500 ms (range 300–890 ms, from first to fourth syllable; Nora et al. 2020).

The environmental sounds were selected from Internet sound libraries. They consisted of 44 high‐quality sound excerpts with corresponding semantic meanings to the spoken words (e.g., chicken clucking, a car engine starting, and guitar music). The environmental sound excerpts were converted to mono sounds with a mean duration of 940 ms (SD = 240 ms), sampling frequency of 44.1 kHz and bit rate of 16 bits using Adobe Audition.

All sounds were subsequently filtered with an 8‐kHz linear low‐pass fast Fourier transform (FFT) filter (Blackman–Harris) and resampled at 16 kHz. The mean amplitudes of the sound excerpts were normalized to have the same RMS intensity and then superimposed using Praat software (Boersma and Weenink 2021). Each superimposition consisted of a spoken word and an environmental sound that were not semantically closely related to each other.

A total of eight different sets of spoken word‐environmental sound combinations were rotated between participants to eliminate the possible confounding effects of specific sound combinations on the results. Furthermore, every word within a specific semantic category was spoken by a different speaker. It is impossible to simultaneously equate both the intensity level of the sound excerpts and their behavioral difficulty. Here we preferred equating the sound intensities (i.e., having the 0 dB SNR within each sound combination) and allowed some variability in the behavioral recognizability. However, the superimposed sounds were behaviorally tested in three volunteers who did not participate in the MEG experiment, to assess the recognizability of both the spoken words and environmental sounds. The semantic meanings of all the sounds were considered identifiable after rearranging some of the sound combinations.

### 2.3 Sound Features

#### 2.3.1 Acoustic Stimulus Features

Two sets of acoustic features that contain time‐varying information (spectrogram and amplitude envelope) were used to model stimulus sounds. The spectrogram represents the sound intensity in various frequency bands over time. The spectrograms were computed using the NSL MATLAB toolbox (Chi et al. 2005) by dividing the sounds into 10‐ms time frames and 128 logarithmically distributed frequency bins ranging from 180 to 7246 Hz.

**FIGURE 1:**
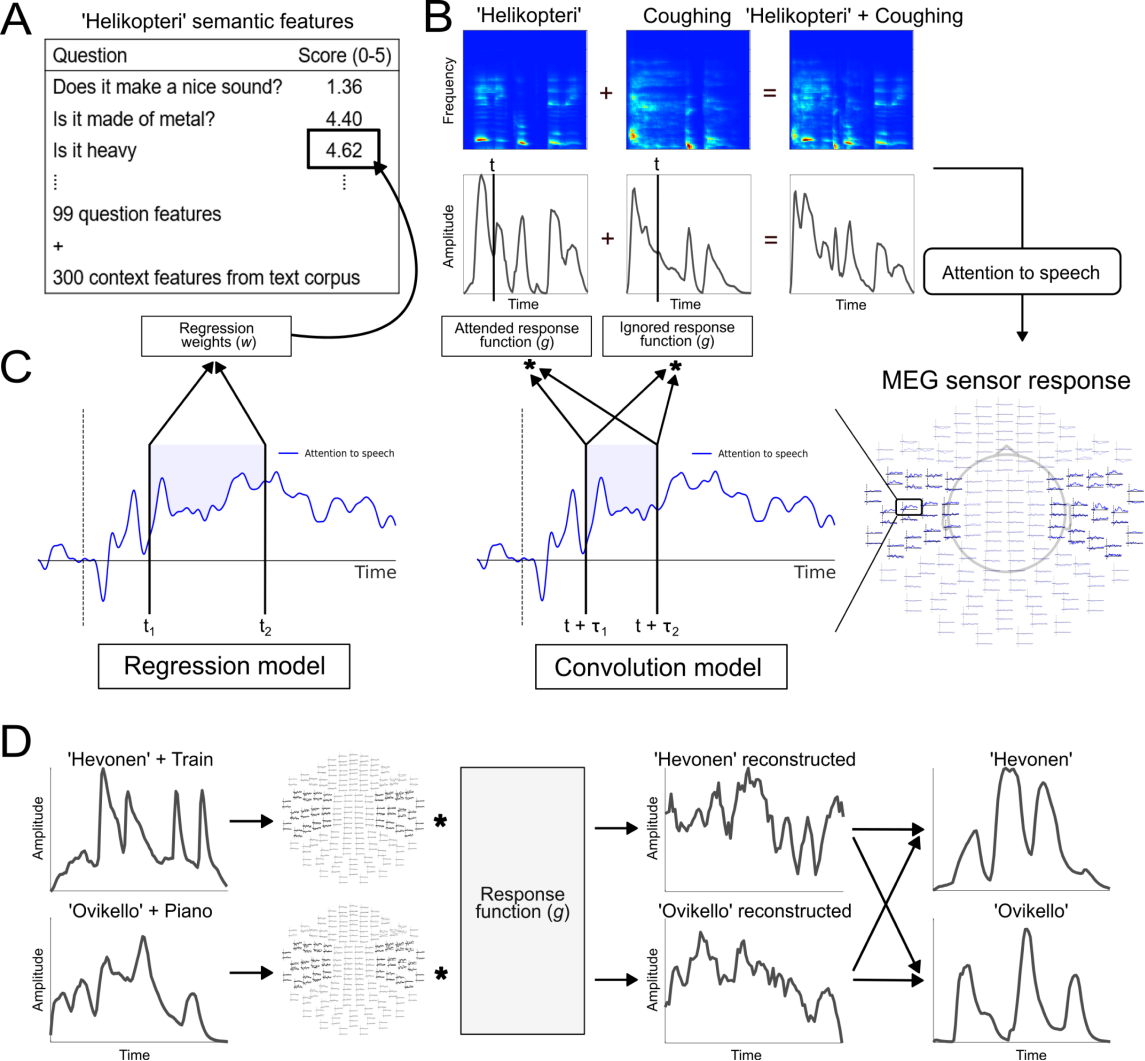
Decoding models for reconstructing acoustic and semantic sound features. (A) Examples of semantic features for the word “helikopteri,” that is, helicopter. (B) Spectrograms (top) and amplitude envelopes (bottom) of the spoken word ‘helikopteri’, the environmental sound of someone coughing, and their superimposition which was presented during the experiment. (C) Illustration of the convolution and linear regression models. The regression model uses the neural response data between $t_{1}$ and $t_{2}$ to decode the semantic features while the convolution model uses neural data between $\left(t+\tau_{1}\right)$ and $\left(t+\tau_{2}\right)$ to decode the acoustic features at time point *t*. Separate convolution models were trained and tested to decode attended and ignored sound excerpts. (D) Procedure for evaluating the performance of the decoding models when reconstructing amplitude envelopes of spoken word excerpts. The MEG responses to two held‐out attended spoken words, here “hevonen,” that is, horse and “ovikello,” that is, doorbell, were convolved with the response function *g* to obtain estimates of the original speech envelopes. The reconstruction was evaluated by comparing the correlation between the reconstructed and original envelopes to the correlation with oppositely labelled envelopes.

The amplitude envelope represents the intensity of the sound over time but lacks spectral information. It was computed by averaging the intensities of all frequency bins in the spectrogram representation for every 10‐ms time window. Example spectrograms and amplitude envelopes for an isolated speech sound, an environmental sound, and their superimposition are shown in Figure 1B.

#### 2.3.2 Semantic Stimulus Features

We also analyzed the processing of the attended and ignored semantic features within the superimposed sounds. The environmental sounds were described with the same set of semantic features as the corresponding spoken/written words. The semantic features of the words were obtained from Nora et al. (2020) and comprised two sets of norms, one acquired through a questionnaire and the other using word co‐occurrences in a large‐scale text corpus. Question norms for the stimulus words were collected with a web‐based survey in which 59 university students answered 99 questions about the semantic properties of each item on a scale from 0 to 5 (Nora et al. 2020).

Corpus statistics were calculated from frequencies of co‐occurrences of words in the immediate neighborhood (five words before and five words after) of each lemmatized stimulus word using a 1.5 billion token Finnish Internet‐derived text corpus (Kanerva and Ginter 2014). Vectors with 300 values were used to describe the corpus statistics of each word, resulting in concatenated semantic feature vectors containing 399 values (Nora et al. 2020).

### 2.4 Experimental Design

Two data sets were recorded for each participant, one in which the participants selectively attended to the spoken word excerpts and one in which they selectively attended to the environmental sound excerpts within the superimposed sounds. Each set contained 20 repetitions of all 44 superimposed sounds presented in a pseudorandomized order such that words spoken by the same speaker or sounds with the same semantic meaning were never presented back‐to‐back. Identical sound combinations were used in both sets. Each data set was recorded in two separate, equally long runs on separate days. The duration of one run was around 17 min with a short break after 8 min. Before each run, the participant was instructed to attend either the speech or environmental sound excerpts. The order of the runs in which the participants attended speech or environmental sounds was counterbalanced between participants to minimize the effect of sound and task familiarity on the results.

Before the first measurement session, the hearing threshold for the experimental stimuli was determined individually for each participant, and the sound intensity was subsequently adjusted to be 60 dB above the hearing threshold. The stimuli were presented using Presentation software (Neurobehavioral Systems Inc., Berkeley, CA, USA) and a panel speaker located in front of the participant. The sounds were presented with an interonset interval between 1.8 and 2.2 s (mean = 2.0 s).

To increase the attention level of the participants and verify that they attended to the intended sound excerpts, they performed a one‐back task in which they lifted a finger each time they identified consecutive sounds with the same semantic meaning (target sounds; approximately 8% of the trials). When participants attended to the spoken word excerpts, the target sounds were superimposed sounds containing the same word as the previous sound but spoken by a different speaker. When attending to the environmental sound excerpts, the target sounds contained a new environmental sound with the same semantic meaning as the previously presented environmental sound (e.g., a dog barking followed by another dog barking). The neural responses to the target sounds were excluded from the final analysis. The response hand was counterbalanced between runs to eliminate its effect on the analysis.

### 2.5 MEG Data Acquisition

Magnetic fields generated by the neural activity were recorded with a 306‐channel neuromagnetometer (Elekta Neuromag TRIUX, MEGIN Oy, Espoo, Finland) inside a magnetically shielded room. The MEG signals were online band‐pass filtered at 0.03–330 Hz and sampled at 1000 Hz. The participant's head position was continuously measured using five head position indicator (HPI) coils attached to the scalp of the participant. The HPI coil locations, three anatomical landmarks (nasion, right and left preauricular points), and multiple extra scalp points were recorded using a 3D digitizer (FASTRAK, Polhemus Inc., Colchester, VT, USA) to enable coregistration between the MEG data and anatomical MRIs. One bipolar electrode pair measuring the electrocardiogram (ECG) signal monitored heartbeat artefacts, while artefacts resulting from blinks and eye movements were monitored with an electrode pair measuring the electrooculogram (EOG) signal.

### 2.6 Anatomical MRI Acquisition

To enable the localization of cortical sources generating the neural responses, anatomical T1‐weighted magnetic resonance images (MRIs) were obtained for 18 out of the 20 participants using a 3 T scanner (MAGNETOM Skyra, Siemens GmbH, Erlangen, Germany) and a 32‐channel head coil. The cortical models for the two participants without individual MRI images were obtained using the fsaverage template head.

### 2.7 MEG Preprocessing and Source Modelling

Spatiotemporal signal space separation (tSSS; Taulu and Simola 2006) implemented in the MaxFilter software (Version 2.2, MEGIN Oy, Espoo, Finland) was applied offline to the MEG data to suppress external magnetic interference and compensate for head movements during the recordings. The head position data was used to transform the head position to the average head position of the recordings. The data was band‐pass filtered at 0.1–40 Hz, and heartbeat and eye movement artefacts were removed using the fastICA algorithm (Hyvärinen 1999). The MEG responses were baseline‐corrected to the 200‐ms interval prior to stimulus onset, and responses corresponding to the same stimuli were subsequently averaged from 300 ms before the stimulus onset to 2000 ms after the stimulus onset.

Individual models of the cortical surface were created from the anatomical MRI images of each participant using FreeSurfer software (Dale et al. 1999; Fischl et al. 1999), and coregistered to the MEG head coordinates based on the three anatomical landmarks and extra scalp points. The lead fields were computed from a single‐compartment boundary element method (BEM) model based on the individual head models. Each hemisphere was covered with roughly 4100 source points distributed over the white matter surface. Source estimates of the evoked MEG responses were obtained using noise‐normalized minimum norm estimates (dynamic statistical parametric mapping [dSPM]; Dale et al. 2000) implemented in the MNE‐Python software (Gramfort et al. 2014), where the noise covariance was estimated from the 300‐ms intervals preceding stimulus onset. The inverse models used a depth weighting exponent of 0.8 to reduce the bias toward superficial sources (Lin et al. 2006), and the sources were loosely constrained to the normal direction of the cortical surface by weighting components tangential to the cortical surface by a factor of 0.2.

The source estimates were morphed to FreeSurfer's cortical surface template (fsaverage) and divided into 273 parcels (126 in the left and 147 in the right hemisphere) based on a subdivided version of the Destrieux atlas (Destrieux et al. 2010). Each parcel contained between 13 and 142 vertices (mean = 69, SD = 22).

### 2.8 Decoding Models

We used a convolution model (Faisal et al. 2015) to reconstruct the time‐varying acoustic features from the MEG evoked responses, while a regression model was used to reconstruct the semantic features. The two models are illustrated in Figure 1C. Importantly, because there may be large interindividual differences in how speech and environmental sounds are processed at the cortical level, we trained the models on data from a single participant and evaluated them on held‐out data from the same participant. The models were always trained to reconstruct features of either the attended or ignored sound excerpts separately, never the superimposed sound features.

Before the final decoding analysis, the MEG signals were downsampled to 100 Hz by averaging blocks of 10 consecutive data points and normalized across all stimuli to have zero mean and unit variance. The MEG signal power per sensor or source‐level PCA component was normalized across time. Each spectrogram frequency band and amplitude envelope was normalized across time, and the semantic feature vectors across all stimuli. This normalization of both MEG data and stimulus features was conducted similarly to Nora et al. (2020), to guarantee that the absolute power at the MEG sensors or within spectrogram frequency bands does not affect the trained models. Instead, model parameters are estimated based on correlations between the variations in the MEG signal power and the variations in the stimulus features.

#### 2.8.1 Convolution Model for Decoding Acoustic Features From MEG Signals

The time‐dependent spectrogram and amplitude envelope features were reconstructed from the evoked MEG responses using a convolution model (Faisal et al. 2015). The model represents a linear mapping between MEG data from time point $\left(t+\tau_{1}\right)$ to $\left(t+\tau_{2}\right)$ and the time‐varying sound features at time point *t*. The convolution model thus decodes the acoustic features at time point *t* using neural responses at time $\left(t+\tau_{1}\right)$ and at successive 10‐ms time points until time ($t+\tau_{2}$). The time lag *τ* at which the MEG responses most accurately track the stimulus features was determined by analyzing 10 consecutive, nonoverlapping time windows of length 40 ms, starting from the window where $\tau_{1}=0$ ms and $\tau_{2}=40$ ms, and ending with the window where $\tau_{1}=360$ ms and $\tau_{2}=400$ ms. Since the time lag is always positive, the model assumes that the neural responses never precede the stimulus in time. The reconstructed time series of the stimulus feature $\hat{s_{f}}$ is described using Equation (1)

(1) $$\hat{s_{f}}\left(t\right)=\sum_{x}\sum_{\tau =\tau_{1}}^{\tau_{2}}g_{f}\left(\tau ,x\right)r\left(t+\tau ,x\right)$$

where $r\left(t,x\right)$ represents the brain response at time t at sensor location x, and $g_{f}\left(\tau ,x\right)$ represents the response function. $\hat{s_{f}}$ represents either the estimated amplitude envelope or the estimate of one single frequency band of the spectrogram.

The mapping between brain responses and acoustic features can be written in matrix notation as $S_{f}=RG_{f}$ where we define $S_{f}\in {\mathbb{R}}^{\left(\left(\mathrm{NT}\right)\times 1\right)}$, $G_{f}\in {\mathbb{R}}^{\left(\left(\mathrm{\tau x}\right)\times 1\right)}$ and the response matrix $R\in {\mathbb{R}}^{\left(\left(\mathrm{NT}\right)\times \left(\mathrm{\tau x}\right)\right)}$. Each row $r_{n}\left(t\right)$ in R represents the MEG response to a spoken word or environmental sound n across all sensors x and all time points between $\left(t+\tau_{1}\right)$ and $\left(t+\tau_{2}\right)$. The unknown function $G_{f}$ is estimated by minimizing the L2‐regularized mean‐squared error between the actual $s_{f}$ and predicted representation $\hat{s_{f}}$ of the stimulus according to Equation (2).

(2) $$\begin{array}{c} \arg\;\mathrm{mi}n_{G_{f}}\sum_{n,t}\left\{s_{f}\left(n,t\right)-\hat{s_{f}}\left(n,t\right)\right\}+\lambda_{f}\sum_{x,\tau }g_{f}{\left(\tau ,x\right)}^{2} \end{array}$$

Minimizing this loss function leads to the maximum‐a posteriori (MAP) estimate for $G_{f}$,

(3) $$\begin{array}{c} \hat{G_{f}}={\left(R^{T}R+\lambda_{f}I\right)}^{-1}R^{T}S_{f} \end{array}$$

where $\lambda_{f}$ is the regularization parameter and I is the identity matrix. This classical MAP estimate is not ideal for MEG studies where the number of conditions are typically small compared to the dimensionality of the neural responses. To solve this problem, the dual (kernel) representation of a convolution model was suggested in (Faisal et al. 2015). Here, the MAP estimate is obtained by replacing the inner product $R^{T}R$ with the corresponding Gram matrix $RR^{T}$, which leads to

(4) $$\begin{array}{c} \hat{G_{f}}=R^{T}{\left(RR^{T}+\lambda_{f}I\right)}^{-1}S_{f} \end{array}$$

To estimate the regularization parameter $\lambda_{f}$, a grid of 23 predefined values between 10 ^− 7^ and 10^7^ was used to find the optimal value that minimizes the leave‐one‐out error within the training data (Faisal et al. 2015). Given the lag parameters $\tau_{1}$ and $\tau_{2}$, the MAP estimates $\hat{G_{f}}$ are used to predict the time‐varying features for the unseen test sounds according to Equation (5).

(5) $$\begin{array}{c} {\hat{s}}_{f,\left\{\text{TEST}\right\}}\left(t\right)=\sum_{x}\sum_{\tau =\tau_{1}}^{\tau =\tau_{2}}g_{f}\left(\tau ,x\right)r_{\left\{\text{TEST}\right\}}\left(t+\tau ,x\right) \end{array}$$

When analyzing MEG sensor‐level data, separate decoding models were trained for MEG data limited to 28 gradiometer sensors located over either the left or right auditory cortex, to reduce the computational load required to train the model. The sensors used in the analysis are highlighted in Figure 1.

To better outline the cortical brain areas important for decoding speech and environmental sounds, the models were also applied to the source‐level data. The source‐level analysis was performed separately within each cortical parcel, by first extracting the time series of the 10 first uncorrelated principal components of each parcel and then training the models on this data. Principal components were used to avoid correlated input features and to remove any effect of model complexity on the decoding results by keeping the number of input features constant for all parcels.

#### 2.8.2 Regression Model for Decoding Semantic Features From MEG Signals

A linear regression model was applied to the MEG data to reconstruct a vector describing semantic features of a sound. The regression model reconstructs each semantic feature separately by finding a linear mapping between the MEG data from $t_{1}$ to $t_{2}$ ms and the semantic feature. The regression model can be described using Equation (6)

(6) $$\begin{array}{c} \hat{s_{f}}=\sum_{x}\sum_{t=t_{1}}^{t_{2}}w_{f}\left(t,x\right)r\left(t,x\right) \end{array}$$

where $r\left(t,x\right)$ represents the brain response at time t at brain location x, and $w_{f}\left(t,x\right)$ represents the regression weights. $\hat{s_{f}}$ represents one element in the semantic feature vector. The optimal weights $w_{f}$ were again learned using ridge regression and the dual representation of the regression problem (Sudre et al. 2012). Here, MEG signals from all 204 gradiometer sensors and the time interval 0–1000 ms was used to train and test the regression model. Additionally, the model was trained and tested using source‐level signals from separate cortical parcels, by again extracting the time series of the 10 first uncorrelated principal components for each parcel.

#### 2.8.3 Performance Evaluation

The ability of the applied models to reconstruct acoustic or semantic sound features based on the MEG responses within each participant was assessed using a leave‐two‐out cross‐validation approach: the model was trained on all but two held‐out sound items ($s_{1}$ and $s_{2}$), similarly to Nora et al. (2020). The learned weights were used to compute predictions of the sound features ($\hat{s_{1}}$ and $\hat{s_{2}}$) based on the neural responses to the two held‐out items. If the similarity (sum of Pearson correlations) between the reconstructed features and the true features was greater than for the opposite labeling, that is, if

(7) $$\begin{array}{c} \text{corr}\left(s_{1},\hat{s_{1}}\right)+\text{corr}\left(s_{2},\hat{s_{2}}\right)>\text{corr}\left(s_{1},\hat{s_{2}}\right)+\text{corr}\left(s_{2},\hat{s_{1}}\right) \end{array}$$

the decoding was considered successful. When evaluating the performance of the convolution model, the durations of the held‐out spectrograms and amplitude envelopes were cut to the length of the shorter one, to eliminate any possible effect of varying feature vector length on the performance of the model. This evaluation approach was repeated for all possible leave‐two‐out combinations, resulting in a total of 946 tests per model and participant. The final decoding accuracy was computed as the percentage of correctly decoded sound pairs.

### 2.9 Statistical Analysis

To assess if the decoding accuracy of a model was above chance level, we compared the decoding accuracy with results obtained from permuted data, similarly to Nora et al. (2020). For the attended sounds, the permutation tests were conducted by running the leave‐two‐out cross‐validation using MEG evoked responses with item labels permuted across sounds. This test was separately performed for each participant, hemisphere, time lag (10 lags), and stimulus type (attended spoken word/environmental sound). In total, 200 different permutation runs were performed for each model, resulting in between‐subject variability of appr. 1% in the obtained participant‐specific significance thresholds and thus considered adequate. The empirical *p* values were computed by counting the percentage of permutation runs in which the decoding accuracy was better than the observed accuracy, separately for each participant.

The empirical, participant‐specific 95% significance thresholds were around 60% (standard deviation [SD] = 0.9%) in the amplitude envelope decoding, 62% (SD = 1.0%) in the spectrogram decoding, and 63% (SD = 1.3%) in the semantic feature decoding. The thresholds were similar for attended spoken words and attended environmental sounds, and they did not depend on the time lag window or hemisphere. The empirical *p* values of all participants were combined using Fisher's method and Bonferroni corrected for multiple comparisons by multiplying the *p* values by the number of time lags (10).

We used two‐tailed Wilcoxon signed‐rank tests to compare the behavioral results when attending to spoken words versus environmental sounds, and the sensor‐level decoding performances for attended spoken words versus environmental sounds and attended versus ignored sounds. For the convolution model, *p* values were separately computed for every time lag and Bonferroni corrected for multiple comparisons.

To compare the source‐level decoding performance for attended spoken words versus environmental sounds, and attended versus ignored spoken words, we used a nonparametric cluster‐based permutation test (Maris and Oostenveld 2007). The continuity‐corrected *Z*‐statistic of the Wilcoxon signed‐rank test was thresholded at *Z* = 1.96 (corresponding to a two‐sided 95% significance threshold) for each parcel, and the *Z*‐scores of spatially adjacent parcels exceeding this threshold were summed together to form the test statistic; two parcels were considered adjacent if they contained connected vertices in the triangular FreeSurfer surface mesh. To obtain multiple comparison‐corrected *p* values, the cluster test statistic was compared to a surrogate null distribution created from 2000 permutations.

To test how differences in cortical tracking between spoken words and environmental sounds relate to attention and stimulus type, we performed a three‐way repeated measures analysis of variance (ANOVA) with factors stimulus type (spoken word or environmental sound), attention (attended or ignored), and lag (80–120, 120–160, 160–200, and 200–240 ms). These time lags were selected based on the study by Nora et al. (2020) which found highest decoding accuracies for spoken words, and the largest decoding differences between spoken words and environmental sounds, at these time lags. The ANOVA analyses were performed using IBM SPSS Statistics 29.0 (IBM Corp., Armonk, NY, USA).

## 3 Results

### 3.1 Behavioral Results

The participants' task was to identify two consecutive sounds with the same semantic meaning within the attended sound stream. As expected, semantic repetitions of spoken words were easier to detect than those of environmental sounds, but participants were generally able to perceive and identify both types of sounds. Participants correctly identified on average 92% (SD = 6%) of the repeated attended words, compared to 74% (SD = 8%) of the repeated attended environmental sounds (Z=3.9, $p=8.8\cdot 10^{-5}$, Wilcoxon signed‐rank test). Additionally, the reaction times were shorter when attending to spoken words (mean = 900 ms, SD = 110 ms) compared to environmental sounds (mean = 1060 ms, SD = 200 ms) (Z=−3.8, $p=1.2\cdot 10^{-4}$, Wilcoxon signed‐rank test). These results are in line with those obtained for individual spoken words and environmental sounds in (Nora et al. 2020).

### 3.2 Neural Responses

**FIGURE 2:**
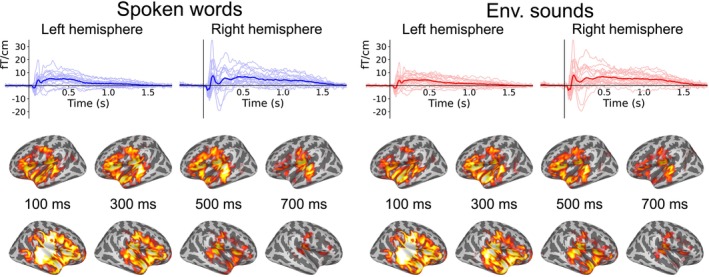
Average sensor (top) and source level (bottom) responses when attending to spoken words and environmental sounds. Each thin line represents the responses of one participant. The sensor responses were averaged across 28 gradiometer sensors located over either the left or right auditory cortex. The source estimates were obtained using dSPM and morphed onto the fsaverage template brain.

The sensor and source‐level brain responses when attending to the spoken word and environmental sound excerpts within the same stimuli are visualized in Figure 2. Attending to both speech and environmental sounds produced clearly discernible auditory evoked responses in both hemispheres, comprising the P50m response (at around 50‐ms latency) followed by a N100m response (at around 100‐ms latency), and a later sustained response from around 200 ms onward. The responses were generally stronger in the right than left hemisphere and varied considerably between subjects, thus highlighting the need for a within‐subject decoding approach.

### 3.3 Acoustic Feature Decoding of Attended Sounds

**FIGURE 3:**
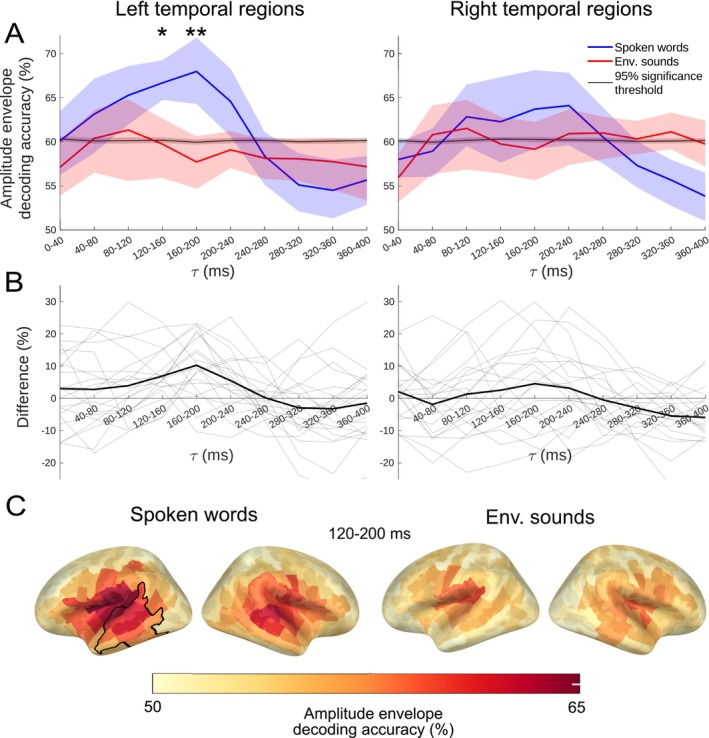
Amplitude envelope decoding accuracies for attended sounds in the left and right temporal regions. (A) Average sensor‐level amplitude envelope decoding accuracies across participants for the attended spoken words and environmental sounds. The stars at 120‐ to 160‐ and 160‐ to 200‐ms lags indicate the time lags where the spoken words were significantly better decoded than the environmental sounds. The 95% significance threshold was averaged across participants and sound categories, and the 95% confidence intervals were obtained by bootstrapping the mean decoding accuracy across participants. (B) The difference in the decoding accuracy between attended spoken words and attended environmental sounds depicted individually for each participant. (C) Source‐level decoding accuracies for each cortical parcel at the time lag of 120–200 ms. The black outline denotes the parcel cluster in which the spoken words were significantly better decoded than the environmental sounds.

First, we trained and tested participant‐specific models to reconstruct amplitude envelopes and spectrogram features of attended spoken words and attended environmental sounds from the MEG responses. The models were trained and tested on the sensor data above the left and right temporal regions separately, and by using source reconstructed data from the separate cortical parcels. Figure 3 shows the results of amplitude envelope decoding for attended spoken words and environmental sounds at the sensor and source levels. For speech, the highest sensor‐level amplitude envelope decoding accuracy was achieved in the left hemisphere at the time lag of 160–200 ms (68.0%, $p=1.6\cdot 10^{-4}$, Bonferroni corrected), and in the right hemisphere at the time lag of 200–240 ms (64.1%, p=0.0014, Bonferroni corrected). For environmental sounds, the highest amplitude envelope decoding accuracy was obtained at the time lag of 80–120 ms in both the left (61.3%, p=0.021, Bonferroni corrected) and right hemisphere (61.5%, p=0.0091, Bonferroni corrected).

In the amplitude envelope analysis, the difference in decoding accuracy between attended spoken words and environmental sounds was statistically significant in the left hemisphere at 120–160 ms (Z=3.1, p=0.02, Wilcoxon signed‐rank test, Bonferroni corrected) and 160–200 ms (Z=3.4, p=0.009, Wilcoxon signed‐rank test, Bonferroni corrected). The difference between decoding accuracies was robust across participants (Figure 3B): at the time lag of 120–160 ms, the decoding accuracy was higher in 16 out of 20 participants for spoken words versus environmental sounds, and at the time lag 160–200 ms, the same was true for 18 out of 20 participants. There were no statistically significant differences between the decoding accuracies in the right temporal regions for any time lag.

Raw correlation values between the original and reconstructed sounds are presented in Figures S1 and S2. The values were consistently higher for speech sounds than for environmental sounds: This difference likely reflects the greater acoustic similarity among speech stimuli and how the similarity is captured in the prominent evoked responses. These observations support the use of leave‐two‐out approach in evaluating the model's ability to discriminate between specific sound pairs, as it is likely to be less influenced by sounds' acoustic similarity.

To investigate which cortical regions contributed to the enhanced spoken word decoding, the corresponding analysis was performed on parcellated source‐level data, using the time‐lag of 120–200 ms. The cortical areas reaching the highest amplitude envelope decoding accuracies were located bilaterally around the auditory cortices (Figure 3C). The highest decoding accuracies for attended spoken words were obtained within the lateral sulci (64.6% in the left hemisphere and 62.7% in the right hemisphere). In addition, the environmental sounds were most accurately decoded in areas around the auditory cortices, reaching a peak decoding accuracy of 60.1% in the left hemisphere and 59.1% in the right hemisphere. The cluster‐based permutation test revealed one parcel cluster, located in the left temporal lobe (Figure 3C), in which the spoken words were significantly better decoded than the environmental sounds (*p* = 0.0035). No significant clusters were found in the right hemisphere.

**FIGURE 4:**
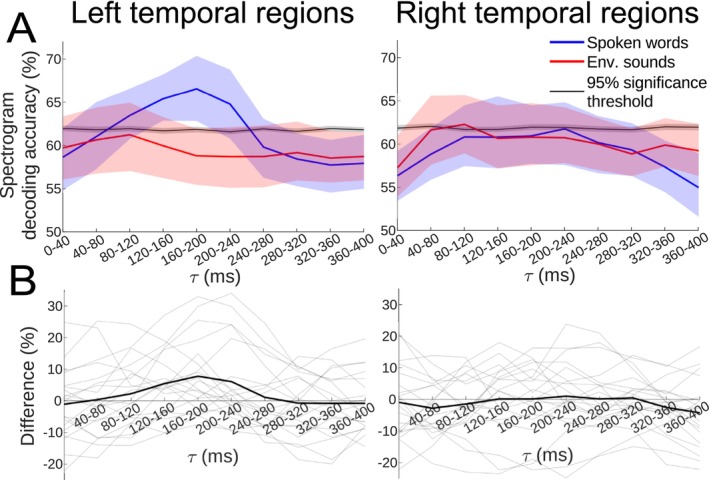
Spectrogram decoding accuracies for attended sounds in the left and right temporal regions. (A) Average spectrogram decoding accuracies across participants for the attended spoken words and environmental sounds. The 95% significance threshold was averaged across participants and sound categories, and the 95% confidence intervals were obtained by bootstrapping the mean decoding accuracies across participants. (B) The difference in decoding accuracy between attended spoken words and attended environmental sounds depicted individually for each participant.

Figure 4 shows the sensor‐level spectrogram decoding accuracies in the right and left temporal regions. The highest spectrogram decoding accuracy for attended speech was again found at the time lag of 160–200 ms in the left temporal region (66.6%, p=0.0067, Bonferroni corrected) and at the time lag of 200–240 ms in the right temporal region (61.8%, p=0.32, Bonferroni corrected). The highest decoding accuracies for environmental sounds were found at the time lag 80–120 ms in both the left (61.2%, p=0.27, Bonferroni corrected) and right hemisphere (62.3%, p=0.21, Bonferroni corrected). We did not observe statistically significant differences between attended spoken words and environmental sounds in the spectrogram decoding analysis, although the spoken word decoding tended to perform better than the environmental sound decoding in the left hemisphere at the same time lag as in the amplitude envelope analysis (160–200 ms; Z=2.5, p=0.13, Wilcoxon signed‐rank test, Bonferroni corrected).

### 3.4 Decoding of Attended Versus Ignored Acoustic Features

**FIGURE 5:**
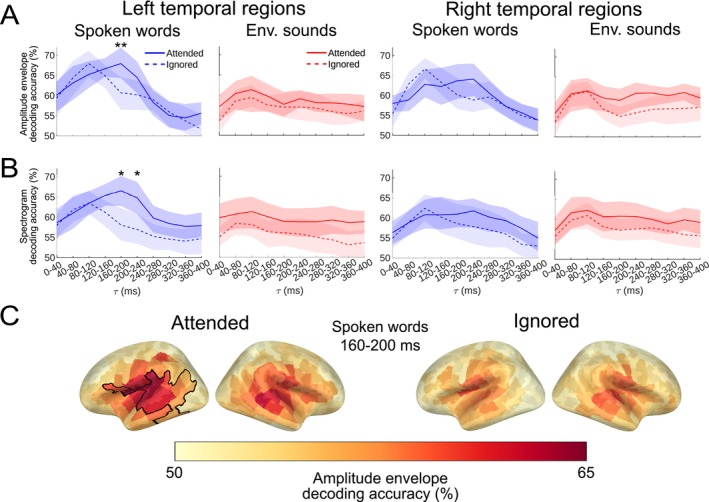
Decoding accuracies for attended and ignored sound excerpts in the left and right temporal regions. (A) The sensor‐level amplitude envelope decoding accuracies. (B) The sensor‐level spectrogram decoding accuracies. The stars at 160–200 and 200–240 ms in the amplitude envelope analysis and 160–200 ms in the spectrogram analysis indicate the time lags where attended sounds were significantly better decoded compared to ignored sounds. (C) Source‐level amplitude envelope decoding accuracies for attended and ignored spoken words at the time lag of 160–200 ms. The black outline denotes the parcel cluster in which the attended spoken words were significantly better decoded than the ignored spoken words.

Next, we trained and tested the models to reconstruct also the ignored sound excerpts, to dissect the contribution of attention and acoustic features on the decoding performance. The sensor‐level decoding results of both the attended and ignored sound envelopes and spectrograms for the left and right temporal regions are presented in Figure 5. In the left hemisphere, the decoding accuracy was higher for the attended than ignored speech envelopes at the time lag of 160–200 ms (Z=3.3, p=0.0089, Wilcoxon signed‐rank test, Bonferroni corrected). Similarly, the attended speech spectrograms were better decoded than ignored speech spectrograms at the lags of 160–200 ms (Z=3.2, p=0.015, Wilcoxon signed‐rank test, Bonferroni corrected) and 200–240 ms (Z=2.8, p=0.045, Wilcoxon signed‐rank test, Bonferroni corrected). In contrast, the decoding accuracies for the attended environmental sounds were never significantly higher than for the ignored environmental sounds, although the average decoding accuracy was higher for the attended sounds at every time lag in both hemispheres. In the right hemisphere, no significant differences were found between the decoding accuracies of attended and ignored sounds for either spoken words or environmental sounds.

To investigate which cortical regions contributed to the enhanced amplitude envelope decoding of attended versus ignored spoken words, the same analysis was performed on parcellated source‐level data using a time‐lag of 160–200 ms. The highest decoding accuracies for attended spoken words were again obtained in regions around the auditory cortices (64.5% in the left hemisphere and 63.0% in the right hemisphere). In addition, the ignored spoken words were most accurately decoded in areas around the auditory cortices, reaching a peak decoding accuracy of 60.0% in the left hemisphere and 60.6% in the right hemisphere. The cluster‐based permutation test revealed one parcel cluster, located in the left temporal and frontal lobe (Figure 5C), where the attended spoken words were significantly better decoded than the ignored spoken words (*p* = 0.0015). No significant clusters were found in the right hemisphere.

We applied a three‐way repeated measures ANOVA to study the effects of the stimulus type (spoken word or environmental sound), attention (attended or ignored), and time lag on the decoding accuracy, and possible interactions between these factors. In the left hemisphere, the ANOVA indicated a significant main effect of stimulus type for both amplitude envelope decoding ($F_{\left(1,19\right)}=24.1$, $p=9.7\cdot 10^{-5}$, $\eta_{p}^{2}=0.56$) and spectrogram decoding ($F_{\left(1,19\right)}=5.16$, p=0.035, $\eta_{p}^{2}=0.21$). Furthermore, ignored spoken word envelopes were better decoded than ignored environmental sound envelopes at lags 80–120 ms (Z=3.02, p=0.025, Wilcoxon signed‐rank test, Bonferroni corrected) and 120–160 ms (Z=3.06, p=0.022, Wilcoxon signed‐rank test, Bonferroni corrected).

The main effect of attention was significant for spectrogram decoding ($F_{\left(1,19\right)}=5.77$, p=0.027, $\eta_{p}^{2}=0.23$) and approached significance for amplitude envelope decoding ($F_{\left(1,19\right)}=4.0$, p=0.06, $\eta_{p}^{2}=0.17$). No significant interactions were found between attention and stimulus type in the amplitude envelope ($F_{\left(1,19\right)}=0.27$, p=0.61, $\eta_{p}^{2}=0.014$) or in the spectrogram analysis ($F_{\left(1,19\right)}=0.87$, p=0.36, $\eta_{p}^{2}=0.044$).

In the right hemisphere, we found a significant main effect of stimulus type (spoken word or environmental sound) for the amplitude envelope decoding ($F_{\left(1,19\right)}=5.52$, p=0.030, $\eta_{p}^{2}=0.23$, ANOVA), but not for the spectrogram decoding. No significant main effect of attention or significant interaction between stimulus type and attention were found for either amplitude envelope or spectrogram decoding.

### 3.5 Semantic Feature Decoding

**FIGURE 6:**
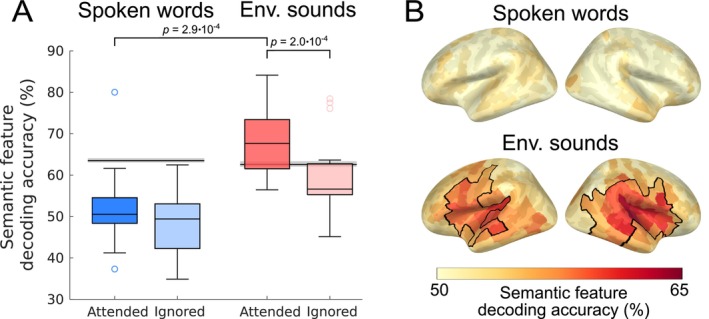
Semantic feature decoding accuracies for spoken words and environmental sounds. (A) Boxplots showing the median decoding accuracy, the 25% and 75% quartiles, outliers, and the minimum and maximum values without outliers. The black lines show the average 95% significance level. (B) Average cortical parcel decoding accuracies for semantic features of attended spoken words and environmental sounds. The black outline denotes the parcel clusters where the environmental sound features were significantly better decoded than the spoken word features.

Figure 6 shows the decoding accuracies of semantic features at both the sensor and the source level. The regression model, applied to sensor‐level signals, achieved an average decoding accuracy of 68.2% ($p=3.1\cdot 10^{-16}$) for semantic features of the attended environmental sounds. The decoding was significantly above chance level (permutation test, *p* < 0.05) in 15/20 participants. For the attended speech sounds, the average semantic feature decoding accuracy was near chance level (52.5%, p=0.23), and it was statistically significant only in 1/20 participants. The two conditions differed significantly from each other (Z=3.6, $p=2.9\cdot 10^{-4}$, Wilcoxon signed‐rank test).

Semantic features of the attended environmental sounds were significantly better decoded compared to the ignored sounds (Z=3.7, $p=2.0\cdot 10^{-4}$, Wilcoxon signed‐rank test), while there was no such difference between the attended versus ignored spoken words. A two‐way repeated measures ANOVA with factors stimulus type (speech or environmental sound) and attention (attended or ignored) indicated a significant main effect of both stimulus type ($F_{\left(1,19\right)}=30.3$, $p=2.7\cdot 10^{-5}$, $\eta_{p}^{2}=0.61$) and attention ($F_{\left(1,19\right)}=18.8$, $p=3.6\cdot 10^{-4}$, $\eta_{p}^{2}=0.50$) on the decoding accuracy, but no significant interaction between them.

At the source level, the highest decoding accuracies for semantic features were observed in the superior temporal and inferior frontal brain regions for attended environmental sounds, while no particular brain region exhibited enhanced decoding accuracy for semantic features of attended spoken words. The cluster‐based permutation test found parcel clusters where the semantic features of environmental sounds were significantly better decoded than the semantic spoken word features in both the left (*p* = 0.003) and right hemisphere (*p* = 0.0005). These clusters were mostly located in the temporal and lateral frontal areas (Figure 6B).

### 3.6 Correlation Between the Behavioral and Decoding Results

Finally, to assess the role of cortical tracking in the on‐going sound recognition task, the Pearson correlation between behavioral task performance and decoding accuracy was computed for both acoustic and semantic feature decoding results of spoken words and environmental sounds. We did not find any significant correlations between the one‐back task performance and decoding of acoustic features in either the left or right temporal areas for any time lag after Bonferroni correcting for multiple comparisons. The one‐back task performance correlated moderately with the decoding of semantic spoken‐word features (ρ=0.48, p=0.034), while there was no significant correlation with the decoding of semantic features of environmental sounds (ρ=−0.17, p=0.49).

## 4 Discussion

In this study, we investigated the time‐locked cortical tracking of speech in conditions that approached natural listening environments. We presented superimposed spoken words and environmental sounds of equal loudness and comparable semantic processing requirements to participants who attended one of the presented sound excerpts at a time. Using MEG and a machine learning approach, we explored whether spoken words are more closely tracked by evoked neural activation than simultaneously presented environmental sounds, and how possible differences in cortical tracking relate to selective attention to sound excerpts versus acoustic feature processing. Our results show that the left temporal brain regions track speech in a more time‐dependent manner than environmental sounds, and that this special mode of time‐locked encoding is shown for speech but not environmental sounds regardless of whether they are attended or ignored. However, attentional top‐down mechanisms do influence speech tracking, as suggested by significantly better decoding of attended than ignored speech in the left hemisphere.

### 4.1 Speech‐Selective Cortical Tracking Extends to Conditions With Overlapping Natural Sounds

While enhanced cortical tracking of speech sounds versus other natural sounds has previously been demonstrated bilaterally for isolated sounds (Nora et al. 2020), our present results show a similar enhancement for speech decoding in more naturalistic listening conditions primarily in the left hemisphere. Although the difference in decoding accuracies between spoken words and environmental sounds was statistically significant only for the amplitude envelope, the speech spectrogram decoding also suggested corresponding differences between speech and environmental sounds and similar attentional effects. The reduction of feature dimensions decreases the complexity of the model and may therefore improve the amplitude envelope decoding results over those of the spectrogram (Nora et al. 2020). Amplitude envelope tracking likely reflects the tracking of spectrotemporal information over multiple frequency bands of the spectrogram and can be thought of as a summary signal of its spectrotemporal modulations, reflecting also phoneme‐level information, not only syllable rhythm (Ding et al. 2014).

We found the highest speech decoding accuracies at time lags of 120–200 ms, consistent with previous results on spoken words presented in isolation (Nora et al. 2020; Nora et al. 2024) or as multiple concurrent speech streams (Fuglsang et al. 2017; O'Sullivan et al. 2015). The peak decoding accuracy observed here, 68% for amplitude envelope decoding in the left hemisphere, is lower than what was observed for isolated spoken words by Nora et al. (2020), suggesting that the addition of environmental sounds in the background partially hampers the cortical tracking of speech. Corresponding degrading effects on the cortical processing of speech have also been observed for vocoded speech and speech embedded in background noise (Ding et al. 2014; Rimmele et al. 2015).

The latency of the strongest decoding accuracies (120–200 ms) coincides with the latency range of prominent auditory evoked components (e.g., the N100m) which are known to be sensitive to rapid changes in acoustic energy. From this perspective, the enhanced tracking of speech compared to environmental sounds could reflect the presence of sharp acoustic edges in speech, such as syllable onsets or phoneme transitions, which phase‐align the neural oscillations (Doelling et al. 2014; Oganian et al. 2023). Environmental sounds, in contrast, often contain less rhythmically structured spectrotemporal information, possibly resulting in weaker and less consistent evoked responses and therefore lower decoding accuracy. No significant correlations were observed between the decoding accuracies of the acoustic features and the behavioral results, suggesting that the decoding results were not driven by differences in attentional load between tasks.

### 4.2 Left‐Lateralized Speech Tracking in Challenging Listening Conditions Highlights Top‐Down Attentional Mechanisms

The cortical areas with the highest speech decoding accuracies were located bilaterally around the auditory cortices, consistent with previous studies indicating that cortical tracking of speech is supported by bilateral neural mechanisms (Brodbeck et al. 2018; Nora et al. 2020). However, we found higher decoding accuracies for spoken word versus environmental sound envelopes only in the left temporal regions, demonstrating that challenging listening conditions may reveal the left‐hemispheric specialization for the segmental processing of speech. This left‐hemispheric enhancement is consistent with earlier imaging studies demonstrating left‐hemispheric specialization for speech and cannot be attributed to signal‐to‐noise differences or generally stronger left‐hemispheric responses, as the auditory evoked responses were generally stronger in the right hemisphere for both stimulus classes.

The left and right auditory cortices are believed to track temporal and spectral speech characteristics at different timescales. According to Poeppel (2003), the processing of fast temporal features (20–50 Hz) lateralizes to the left auditory cortex, while the right auditory cortex would integrate and process primarily slow temporal features at 4–10 Hz. The speech envelope is characterized mainly by slow amplitude modulations that correspond to the syllabic rate (approximately 3–5 Hz). Consistent with this asymmetry, several studies have reported stronger neural tracking of the speech envelope in the right hemisphere (Abrams et al. 2008; Ding and Simon 2012a; Luo and Poeppel 2007).

Nevertheless, our results showed significantly higher decoding accuracies for spoken word versus environmental sound envelopes only in the left hemisphere. Furthermore, only the left temporal regions showed significantly higher decoding accuracies for attended speech compared to ignored speech, suggesting that the observed left‐lateralization may be at least partially driven by enhanced top‐down processing in the present task, in line with the view that cortical tracking is largely shaped by top‐down processes (Giraud and Poeppel 2012; Schroeder and Lakatos 2009). Although the effect of attention was only significant for spoken words, we did not find any significant interaction between attention and sound type in the ANOVA analysis. Since the ANOVA was applied over a wide time window ranging from 80‐ to 240‐ms latency, it is possible that the analysis could not identify effects only present in narrow time windows and therefore did not reach statistical significance.

Previous neuroimaging research has demonstrated that the effect of selective attention to a certain speech stream is lateralized toward the left hemisphere (Power et al. 2012), possibly supported by frontal and motor areas known to modulate the oscillatory activity of the auditory cortices particularly in the left hemisphere (Assaneo et al. 2019; Park et al. 2015). This top‐down control is believed to relate to predicting upcoming periodic and aperiodic events from, for example, acoustic, syntactic and semantic cues in the incoming sound stream (Rimmele et al. 2018), by aligning neural oscillations with fluctuations in the speech signal (Assaneo et al. 2019; Park et al. 2015; Park et al. 2018). Such an experience‐dependent processing mode may be particularly important for speech perception in noisy listening conditions (Sohoglu and Davis 2016). This interpretation is also supported by earlier results, in which the coupling between slow delta band oscillations (~0.5 Hz) and slow amplitude modulations in attended continuous speech became left‐lateralized when background noise was added (Vander Ghinst et al. 2016). Here we show a similar effect of attention on the cortical tracking of single words in evoked responses, demonstrating the importance of such tracking for segmental word‐level processing in challenging listening conditions.

No significant differences were found between the attended and ignored sound streams in the environmental sound analysis. Furthermore, our results indicated similar environmental sound decoding accuracies across the hemispheres, differing from the leftward asymmetry present for the spoken‐word decoding. This supports the theory that environmental sound processing mainly reflects bottom‐up acoustics‐driven mechanisms and would therefore be more bilateral or right‐lateralized than speech processing (Assaneo et al. 2019), although specific listening conditions can significantly influence the observed lateralization. For example, ambiguous sounds elicit stronger responses in the left hemisphere when perceived as speech (Dehaene‐Lambertz et al. 2005; Möttönen et al. 2006), and attention to phonological content within sounds increasingly activates the left temporal regions (Yoncheva et al. 2014). These observations suggest that while environmental sounds are generally processed bilaterally, left‐hemispheric recruitment can emerge under conditions that encourage speech‐like interpretation or phonological analysis.

Bottom‐up modulated cortical tracking also seems important for processing speech. Our results indicated a significant main effect of the stimulus type in the amplitude envelope decoding analysis (both hemispheres) and in the spectrogram decoding analysis (left hemisphere). Thus, both attended and ignored speech was better decoded than the environmental sound counterparts. This result seems to be partially driven by the high decoding accuracy for ignored speech at 80‐ to 120‐ms latency, indicating that bottom‐up modulated processes take place in an earlier time window compared to top‐down driven processes, in line with previous studies (Brodbeck et al. 2020; Karunathilake et al. 2023).

### 4.3 Stronger Semantic Decoding of Environmental Than Speech Sounds May Reflect Greater Processing Demands

The behavioral recognition of semantically similar back‐to‐back environmental sounds was more difficult during the experiment than recognition of consecutive speech sounds with the same meanings. However, the accuracies in the behavioral task were in line with previous results obtained for the same speech and environmental sounds presented in isolation (94% for spoken words, 78% for environmental sounds; Nora et al. 2020), suggesting that neither the spoken words nor the environmental sounds were excessively masked by the applied superimposition.

Interestingly, the semantic features of attended environmental sounds were better decoded than those of the attended speech sounds, consistent with previous studies (Nora et al. 2020; Nora et al. 2024; see also Simanova et al. 2010). We suggest that this reflects the nature of the stimulus categories: Semantic decoding may be stronger for environmental sounds because their semantic categories are less well defined and less overlearned than those of spoken words. Consequently, resolving semantic similarity between consecutive environmental sounds likely requires more extensive cortical processing, leading to more discriminable neural representations. This could, in turn, increase decoding accuracy at the neural level, even if behavioral performance is lower. This interpretation agrees with the significantly higher semantic decoding accuracy of environmental sounds than spoken words also in the unattended condition.

Individual differences in speech semantic decoding accuracy correlated with behavioral performance, suggesting that meaningful neural representations of speech semantics were also present but varied across participants: those with more robust decodable representations of speech semantics tended to perform better in the behavioral task. Together, the stimulus category‐level differences and the brain–behavior correlations suggest that decodability reflects not only cortical representation strength but also the degree to which the semantic features within a stimulus category are discriminable and variable across trials.

## 5 Conclusions

In conclusion, our results show that attended spoken words are more accurately tracked in time than other attended natural sounds in the left temporal regions at around 120‐ to 200‐ms time delay between the sound and neuronal activation also in conditions that resemble naturalistic environments. The enhanced cortical tracking appears to reflect both bottom‐up processing of speech‐specific acoustic features and top‐down mechanisms when attending to speech. The present study was conducted using isolated sound combinations which allowed us to control acoustic and semantic properties more accurately than with continuous speech. Furthermore, the present question—how speech and environmental sounds are decoded concurrently—benefitted from minimizing any higher‐level contextual and predictive effects inherent to longer auditory scenes. However, further studies should address also longer auditory scenes, as well as multimodal processing. Superimposed speech and environmental sounds could be studied, for example, by using temporal response functions (TRFs), which have previously revealed differences in the cortical tracking of attended and ignored speech (Akram et al. 2017; Fiedler et al. 2019), and could reveal possible encoding differences also for continuous speech and environmental sounds (Crosse et al. 2021).

## Author Contributions

**Jesper Edström:** data curation, formal analysis, investigation, methodology, software, visualization, writing – original draft, writing – review and editing. **Anni Nora:** conceptualization, writing – review and editing. **Oona Rinkinen:** methodology, software, writing – review and editing. **Riitta Salmelin:** conceptualization, funding acquisition, writing – review and editing. **Hanna Renvall:** conceptualization, data curation, funding acquisition, investigation, supervision, writing – review and editing.

## Funding

This work was supported by the Sigrid Juséliuksen Säätiö, Finnish Ministry of Education and Culture's Doctoral Pilot for Mathematics of Sensing, Imaging and Modelling, Research Council of Finland (321460, 332622, 355407, and 355409), and the Flagship of Advanced Mathematics for Sensing Imaging and Modeling (359181).

## Ethics Statement

The study obtained a prior ethical statement from the Aalto University Research Ethics Committee, and the participants gave their written consent before the measurements.

## Conflicts of Interest

The authors declare no conflicts of interest.

## Supporting information


**Figure S1:** Raw correlation values between the original and reconstructed sounds in the sensor‐level amplitude envelope decoding analysis. The values corresponding to the shortest (0–40 ms) and longest (360–400 ms) latencies are marked, as well as the statistically significant latencies of 120–160 and 160–200 ms.
**Figure S2:** Raw correlation values between the original and reconstructed sounds in the sensor‐level spectrogram decoding analysis. The values corresponding to the shortest (0–40 ms) and longest (360–400 ms) latencies are marked, as well as the statistically significant latencies of 160–200 and 200–240 ms.

## Data Availability

The MEG data cannot be made publicly available due to ethical restrictions imposed by the research ethics committee statement. Relevant derived and pseudonymized data supporting the findings of this study can be shared upon reasonable request and with permission of the research ethics committee for researchers aiming to reproduce the results. Custom code used in the decoding analysis is made openly available at Gitlab (https://version.aalto.fi/gitlab/biomag‐pipelines/cortical_tracking_decoding).

## References

[ejn70598-bib-0001] Abrams, D. A. , T. Nicol , S. Zecker , and N. Kraus . 2008. “Right‐Hemisphere Auditory Cortex Is Dominant for Coding Syllable Patterns in Speech.” Journal of Neuroscience 28, no. 15: 3958–3965. 10.1523/JNEUROSCI.0187-08.2008. 18400895 PMC2713056

[ejn70598-bib-0002] Ahissar, E. , S. Nagarajan , M. Ahissar , A. Protopapas , H. Mahncke , and M. M. Merzenich . 2001. “Speech Comprehension Is Correlated With Temporal Response Patterns Recorded From Auditory Cortex.” Proceedings of the National Academy of Sciences 98, no. 23: 13367–13372. 10.1073/pnas.201400998. PMC60877 11698688

[ejn70598-bib-0003] Akram, S. , J. Z. Simon , and B. Babadi . 2017. “Dynamic Estimation of the Auditory Temporal Response Function From MEG in Competing‐Speaker Environments.” IEEE Transactions on Biomedical Engineering 64, no. 8: 1896–1905. 10.1109/TBME.2016.2628884. 28113290 PMC5568457

[ejn70598-bib-0004] Alain, C. , S. R. Arnott , and T. W. Picton . 2001. “Bottom–up and Top–Down Influences on Auditory Scene Analysis: Evidence From Event‐Related Brain Potentials.” Journal of Experimental Psychology: Human Perception and Performance 27, no. 5: 1072–1089. 10.1037/0096-1523.27.5.1072. 11642696

[ejn70598-bib-0005] Assaneo, M. F. , J. M. Rimmele , J. Orpella , P. Ripollés , R. de Diego‐Balaguer , and D. Poeppel . 2019. “The Lateralization of Speech‐Brain Coupling Is Differentially Modulated by Intrinsic Auditory and Top‐Down Mechanisms.” Frontiers in Integrative Neuroscience 13: 28. 10.3389/fnint.2019.00028. 31379527 PMC6650591

[ejn70598-bib-0006] Boersma, P. , and D. Weenink . 2021. “Praat: Doing Phonetics by Computer [Computer Program] (Version 6.1.48).” https://www.fon.hum.uva.nl/praat/.

[ejn70598-bib-0007] Bregman, A. S. 1990. Auditory Scene Analysis: The Perceptual Organization of Sound. MIT Press. 10.7551/mitpress/1486.001.0001.

[ejn70598-bib-0008] Brodbeck, C. , A. Jiao , L. E. Hong , and J. Z. Simon . 2020. “Neural Speech Restoration at the Cocktail Party: Auditory Cortex Recovers Masked Speech of Both Attended and Ignored Speakers.” PLoS Biology 18, no. 10: e3000883. 10.1371/journal.pbio.3000883. 33091003 PMC7644085

[ejn70598-bib-0009] Brodbeck, C. , A. Presacco , and J. Z. Simon . 2018. “Neural Source Dynamics of Brain Responses to Continuous Stimuli: Speech Processing From Acoustics to Comprehension.” NeuroImage 172: 162–174. 10.1016/j.neuroimage.2018.01.042. 29366698 PMC5910254

[ejn70598-bib-0010] Chi, T. , P. Ru , and S. A. Shamma . 2005. “Multiresolution Spectrotemporal Analysis of Complex Sounds.” Journal of the Acoustical Society of America 118, no. 2: 887–906. 10.1121/1.1945807. 16158645

[ejn70598-bib-0011] Crosse, M. J. , N. J. Zuk , G. M. Di Liberto , A. R. Nidiffer , S. Molholm , and E. C. Lalor . 2021. “Linear Modeling of Neurophysiological Responses to Speech and Other Continuous Stimuli: Methodological Considerations for Applied Research.” Frontiers in Neuroscience 15: 705621. 10.3389/fnins.2021.705621. 34880719 PMC8648261

[ejn70598-bib-0012] Dale, A. M. , B. Fischl , and M. I. Sereno . 1999. “Cortical Surface‐Based Analysis: I. Segmentation and Surface Reconstruction.” NeuroImage 9, no. 2: 179–194. 10.1006/nimg.1998.0395. 9931268

[ejn70598-bib-0013] Dale, A. M. , A. K. Liu , B. R. Fischl , et al. 2000. “Dynamic Statistical Parametric Mapping: Combining fMRI and MEG for High‐Resolution Imaging of Cortical Activity.” Neuron 26, no. 1: 55–67. 10.1016/S0896-6273(00)81138-1. 10798392

[ejn70598-bib-0014] Dehaene‐Lambertz, G. , C. Pallier , W. Serniclaes , L. Sprenger‐Charolles , A. Jobert , and S. Dehaene . 2005. “Neural Correlates of Switching From Auditory to Speech Perception.” NeuroImage 24, no. 1: 21–33. 10.1016/j.neuroimage.2004.09.039. 15588593

[ejn70598-bib-0015] Desai, M. , J. Holder , C. Villarreal , N. Clark , B. Hoang , and L. S. Hamilton . 2021. “Generalizable EEG Encoding Models With Naturalistic Audiovisual Stimuli.” Journal of Neuroscience 41, no. 43: 8946–8962. 10.1523/JNEUROSCI.2891-20.2021. 34503996 PMC8549533

[ejn70598-bib-0016] Destrieux, C. , B. Fischl , A. Dale , and E. Halgren . 2010. “Automatic Parcellation of Human Cortical Gyri and Sulci Using Standard Anatomical Nomenclature.” NeuroImage 53, no. 1: 1–15. 10.1016/j.neuroimage.2010.06.010. 20547229 PMC2937159

[ejn70598-bib-0017] Ding, N. , M. Chatterjee , and J. Z. Simon . 2014. “Robust Cortical Entrainment to the Speech Envelope Relies on the Spectro‐Temporal Fine Structure.” NeuroImage 88: 41–46. 10.1016/j.neuroimage.2013.10.054. 24188816 PMC4222995

[ejn70598-bib-0018] Ding, N. , and J. Z. Simon . 2012a. “Neural Coding of Continuous Speech in Auditory Cortex During Monaural and Dichotic Listening.” Journal of Neurophysiology 107, no. 1: 78–89. 10.1152/jn.00297.2011. 21975452 PMC3570829

[ejn70598-bib-0019] Ding, N. , and J. Z. Simon . 2012b. “Emergence of Neural Encoding of Auditory Objects While Listening to Competing Speakers.” Proceedings of the National Academy of Sciences 109, no. 29: 11854–11859. 10.1073/pnas.1205381109. PMC3406818 22753470

[ejn70598-bib-0020] Ding, N. , and J. Z. Simon . 2014. “Cortical Entrainment to Continuous Speech: Functional Roles and Interpretations.” Frontiers in Human Neuroscience 8: 311. 10.3389/fnhum.2014.00311. 24904354 PMC4036061

[ejn70598-bib-0021] Doelling, K. B. , L. H. Arnal , O. Ghitza , and D. Poeppel . 2014. “Acoustic Landmarks Drive Delta–Theta Oscillations to Enable Speech Comprehension by Facilitating Perceptual Parsing.” NeuroImage 85: 761–768. 10.1016/j.neuroimage.2013.06.035. 23791839 PMC3839250

[ejn70598-bib-0022] Ershaid, H. , M. Lizarazu , D. McLaughlin , et al. 2024. “Contributions of Listening Effort and Intelligibility to Cortical Tracking of Speech in Adverse Listening Conditions.” Cortex 172: 54–71. 10.1016/j.cortex.2023.11.018. 38215511

[ejn70598-bib-0023] Faisal, A. , A. Nora , J. Seol , H. Renvall , and R. Salmelin . 2015. “Kernel Convolution Model for Decoding Sounds From Time‐Varying Neural Responses.” 2015 International Workshop on Pattern Recognition in NeuroImaging. 49–52. 10.1109/PRNI.2015.10.

[ejn70598-bib-0024] Fiedler, L. , M. Wöstmann , S. K. Herbst , and J. Obleser . 2019. “Late Cortical Tracking of Ignored Speech Facilitates Neural Selectivity in Acoustically Challenging Conditions.” NeuroImage 186: 33–42. 10.1016/j.neuroimage.2018.10.057. 30367953

[ejn70598-bib-0025] Fischl, B. , M. I. Sereno , and A. M. Dale . 1999. “Cortical Surface‐Based Analysis: II: Inflation, Flattening, and a Surface‐Based Coordinate System.” NeuroImage 9, no. 2: 195–207. 10.1006/nimg.1998.0396. 9931269

[ejn70598-bib-0026] Fuglsang, S. A. , T. Dau , and J. Hjortkjær . 2017. “Noise‐Robust Cortical Tracking of Attended Speech in Real‐World Acoustic Scenes.” NeuroImage 156: 435–444. 10.1016/j.neuroimage.2017.04.026. 28412441

[ejn70598-bib-0027] Giraud, A.‐L. , and D. Poeppel . 2012. “Cortical Oscillations and Speech Processing: Emerging Computational Principles and Operations.” Nature Neuroscience 15, no. 4: 511–517. 10.1038/nn.3063. 22426255 PMC4461038

[ejn70598-bib-0028] Gramfort, A. , M. Luessi , E. Larson , et al. 2014. “MNE Software for Processing MEG and EEG Data.” NeuroImage 86: 446–460. 10.1016/j.neuroimage.2013.10.027. 24161808 PMC3930851

[ejn70598-bib-0029] Horton, C. , M. D'Zmura , and R. Srinivasan . 2013. “Suppression of Competing Speech Through Entrainment of Cortical Oscillations.” Journal of Neurophysiology 109, no. 12: 3082–3093. 10.1152/jn.01026.2012. 23515789 PMC3680812

[ejn70598-bib-0030] Hyvärinen, A. 1999. “Fast and Robust Fixed‐Point Algorithms for Independent Component Analysis.” IEEE Transactions on Neural Networks 10, no. 3: 626–634. 10.1109/72.761722. 18252563

[ejn70598-bib-0031] Kanerva, J. , and F. Ginter . 2014. “Post‐Hoc Manipulations of Vector Space Models With Application to Semantic Role Labeling.” Proceedings of the 2nd Workshop on Continuous Vector Space Models and Their Compositionality (CVSC). 1–10. 10.3115/v1/W14-1501.

[ejn70598-bib-0032] Karunathilake, I. M. D. , J. P. Kulasingham , and J. Z. Simon . 2023. “Neural Tracking Measures of Speech Intelligibility: Manipulating Intelligibility While Keeping Acoustics Unchanged.” Proceedings of the National Academy of Sciences 120, no. 49: e2309166120. 10.1073/pnas.2309166120. PMC10710032 38032934

[ejn70598-bib-0033] Kerlin, J. R. , A. J. Shahin , and L. M. Miller . 2010. “Attentional Gain Control of Ongoing Cortical Speech Representations in a “Cocktail Party”.” Journal of Neuroscience 30, no. 2: 620–628. 10.1523/JNEUROSCI.3631-09.2010. 20071526 PMC2832933

[ejn70598-bib-0034] Khalighinejad, B. , J. L. Herrero , A. D. Mehta , and N. Mesgarani . 2019. “Adaptation of the Human Auditory Cortex to Changing Background Noise.” Nature Communications 10, no. 1: 2509. 10.1038/s41467-019-10611-4. PMC6555798 31175304

[ejn70598-bib-0035] Kong, Y.‐Y. , A. Mullangi , and N. Ding . 2014. “Differential Modulation of Auditory Responses to Attended and Unattended Speech in Different Listening Conditions.” Hearing Research 316: 73–81. 10.1016/j.heares.2014.07.009. 25124153 PMC4194271

[ejn70598-bib-0036] Lin, F.‐H. , T. Witzel , S. P. Ahlfors , S. M. Stufflebeam , J. W. Belliveau , and M. S. Hämäläinen . 2006. “Assessing and Improving the Spatial Accuracy in MEG Source Localization by Depth‐Weighted Minimum‐Norm Estimates.” NeuroImage 31, no. 1: 160–171. 10.1016/j.neuroimage.2005.11.054. 16520063

[ejn70598-bib-0037] Luo, H. , and D. Poeppel . 2007. “Phase Patterns of Neuronal Responses Reliably Discriminate Speech in Human Auditory Cortex.” Neuron 54, no. 6: 1001–1010. 10.1016/j.neuron.2007.06.004. 17582338 PMC2703451

[ejn70598-bib-0038] Maris, E. , and R. Oostenveld . 2007. “Nonparametric Statistical Testing of EEG‐ and MEG‐Data.” Journal of Neuroscience Methods 164, no. 1: 177–190. 10.1016/j.jneumeth.2007.03.024. 17517438

[ejn70598-bib-0039] Mesgarani, N. , and E. F. Chang . 2012. “Selective Cortical Representation of Attended Speaker in Multi‐Talker Speech Perception.” Nature 485, no. 7397: 233–236. 10.1038/nature11020. 22522927 PMC3870007

[ejn70598-bib-0040] Mirkovic, B. , S. Debener , M. Jaeger , and M. D. Vos . 2015. “Decoding the Attended Speech Stream With Multi‐Channel EEG: Implications for Online, Daily‐Life Applications.” Journal of Neural Engineering 12, no. 4: 046007. 10.1088/1741-2560/12/4/046007. 26035345

[ejn70598-bib-0041] Möttönen, R. , G. A. Calvert , I. P. Jääskeläinen , et al. 2006. “Perceiving Identical Sounds as Speech or Nonspeech Modulates Activity in the Left Posterior Superior Temporal Sulcus.” NeuroImage 30, no. 2: 563–569. 10.1016/j.neuroimage.2005.10.002. 16275021

[ejn70598-bib-0042] Nora, A. , A. Faisal , J. Seol , H. Renvall , E. Formisano , and R. Salmelin . 2020. “Dynamic Time‐Locking Mechanism in the Cortical Representation of Spoken Words.” eNeuro 7, no. 4: ENEURO.0475‐19.2020. 10.1523/ENEURO.0475-19.2020. PMC7470935 32513662

[ejn70598-bib-0043] Nora, A. , O. Rinkinen , H. Renvall , et al. 2024. “Impaired Cortical Tracking of Speech in Children With Developmental Language Disorder.” Journal of Neuroscience 44, no. 22: e2048232024. 10.1523/JNEUROSCI.2048-23.2024. 38589232 PMC11140678

[ejn70598-bib-0044] Obleser, J. , and C. Kayser . 2019. “Neural Entrainment and Attentional Selection in the Listening Brain.” Trends in Cognitive Sciences 23, no. 11: 913–926. 10.1016/j.tics.2019.08.004. 31606386

[ejn70598-bib-0045] Oganian, Y. , K. Kojima , A. Breska , et al. 2023. “Phase Alignment of Low‐Frequency Neural Activity to the Amplitude Envelope of Speech Reflects Evoked Responses to Acoustic Edges, Not Oscillatory Entrainment.” Journal of Neuroscience 43, no. 21: 3909–3921. 10.1523/JNEUROSCI.1663-22.2023. 37185238 PMC10218004

[ejn70598-bib-0046] O'Sullivan, J. A. , A. J. Power , N. Mesgarani , et al. 2015. “Attentional Selection in a Cocktail Party Environment Can Be Decoded From Single‐Trial EEG.” Cerebral Cortex 25, no. 7: 1697–1706. 10.1093/cercor/bht355. 24429136 PMC4481604

[ejn70598-bib-0047] Park, H. , R. A. Ince , P. G. Schyns , G. Thut , and J. Gross . 2015. “Frontal Top‐Down Signals Increase Coupling of Auditory Low‐Frequency Oscillations to Continuous Speech in Human Listeners.” Current Biology 25, no. 12: 1649–1653. 10.1016/j.cub.2015.04.049. 26028433 PMC4503802

[ejn70598-bib-0048] Park, H. , G. Thut , and J. Gross . 2018. “Predictive Entrainment of Natural Speech Through Two Fronto‐Motor Top‐Down Channels.” Language, Cognition and Neuroscience 35, no. 6: 739–751. 10.1080/23273798.2018.1506589. 32939354 PMC7446042

[ejn70598-bib-0049] Peelle, J. E. , J. Gross , and M. H. Davis . 2013. “Phase‐Locked Responses to Speech in Human Auditory Cortex Are Enhanced During Comprehension.” Cerebral Cortex 23, no. 6: 1378–1387. 10.1093/cercor/bhs118. 22610394 PMC3643716

[ejn70598-bib-0050] Pichora‐Fuller, M. K. , B. A. Schneider , and M. Daneman . 1995. “How Young and Old Adults Listen to and Remember Speech in Noise.” Journal of the Acoustical Society of America 97, no. 1: 593–608. 10.1121/1.412282. 7860836

[ejn70598-bib-0051] Poeppel, D. 2003. “The Analysis of Speech in Different Temporal Integration Windows: Cerebral Lateralization as ‘Asymmetric Sampling in Time’.” Speech Communication 41, no. 1: 245–255. 10.1016/S0167-6393(02)00107-3.

[ejn70598-bib-0052] Power, A. J. , J. J. Foxe , E.‐J. Forde , R. B. Reilly , and E. C. Lalor . 2012. “At What Time Is the Cocktail Party? A Late Locus of Selective Attention to Natural Speech.” European Journal of Neuroscience 35, no. 9: 1497–1503. 10.1111/j.1460-9568.2012.08060.x. 22462504

[ejn70598-bib-0053] Renvall, H. , J. Seol , R. Tuominen , B. Sorger , L. Riecke , and R. Salmelin . 2021. “Selective Auditory Attention Within Naturalistic Scenes Modulates Reactivity to Speech Sounds.” European Journal of Neuroscience 54, no. 10: 7626–7641. 10.1111/ejn.15504. 34697833 PMC9298413

[ejn70598-bib-0054] Rimmele, J. M. , B. Morillon , D. Poeppel , and L. H. Arnal . 2018. “Proactive Sensing of Periodic and Aperiodic Auditory Patterns.” Trends in Cognitive Sciences 22, no. 10: 870–882. 10.1016/j.tics.2018.08.003. 30266147

[ejn70598-bib-0055] Rimmele, J. M. , E. Zion Golumbic , E. Schröger , and D. Poeppel . 2015. “The Effects of Selective Attention and Speech Acoustics on Neural Speech‐Tracking in a Multi‐Talker Scene.” Cortex 68: 144–154. 10.1016/j.cortex.2014.12.014. 25650107 PMC4475476

[ejn70598-bib-0056] Schroeder, C. E. , and P. Lakatos . 2009. “Low‐Frequency Neuronal Oscillations as Instruments of Sensory Selection.” Trends in Neurosciences 32, no. 1: 9–18. 10.1016/j.tins.2008.09.012. 19012975 PMC2990947

[ejn70598-bib-0057] Simanova, I. , M. van Gerven , R. Oostenveld , and P. Hagoort . 2010. “Identifying Object Categories From Event‐Related EEG: Toward Decoding of Conceptual Representations.” PLoS ONE 5, no. 12: e14465. 10.1371/journal.pone.0014465. 21209937 PMC3012689

[ejn70598-bib-0058] Sohoglu, E. , and M. H. Davis . 2016. “Perceptual Learning of Degraded Speech by Minimizing Prediction Error.” Proceedings of the National Academy of Sciences 113, no. 12: E1747–E1756. 10.1073/pnas.1523266113. PMC4812728 26957596

[ejn70598-bib-0059] Sudre, G. , D. Pomerleau , M. Palatucci , et al. 2012. “Tracking Neural Coding of Perceptual and Semantic Features of Concrete Nouns.” NeuroImage 62, no. 1: 451–463. 10.1016/j.neuroimage.2012.04.048. 22565201 PMC4465409

[ejn70598-bib-0060] Taulu, S. , and J. Simola . 2006. “Spatiotemporal Signal Space Separation Method for Rejecting Nearby Interference in MEG Measurements.” Physics in Medicine & Biology 51, no. 7: 1759–1768. 10.1088/0031-9155/51/7/008. 16552102

[ejn70598-bib-0061] Vander Ghinst, M. , M. Bourguignon , M. Op de Beeck , et al. 2016. “Left Superior Temporal Gyrus Is Coupled to Attended Speech in a Cocktail‐Party Auditory Scene.” Journal of Neuroscience 36, no. 5: 1596–1606. 10.1523/JNEUROSCI.1730-15.2016. 26843641 PMC6601992

[ejn70598-bib-0062] Wang, L. , E. X. Wu , and F. Chen . 2020. “Robust EEG‐Based Decoding of Auditory Attention With High‐RMS‐Level Speech Segments in Noisy Conditions.” Frontiers in Human Neuroscience 14: 557534. 10.3389/fnhum.2020.557534. 33132874 PMC7576187

[ejn70598-bib-0063] Yoncheva, Y. , U. Maurer , J. D. Zevin , and B. D. McCandliss . 2014. “Selective Attention to Phonology Dynamically Modulates Initial Encoding of Auditory Words Within the Left Hemisphere.” NeuroImage 97: 262–270. 10.1016/j.neuroimage.855-2014.04.006. 24746955 PMC4414015

[ejn70598-bib-0064] Zekveld, A. A. , D. J. Heslenfeld , J. M. Festen , and R. Schoonhoven . 2006. “Top–Down and Bottom–up Processes in Speech Comprehension.” NeuroImage 32, no. 4: 1826–1836. 10.1016/j.neuroimage.2006.04.199. 16781167

[ejn70598-bib-0065] Zion Golumbic, E. M. , N. Ding , S. Bickel , et al. 2013. “Mechanisms Underlying Selective Neuronal Tracking of Attended Speech at a “Cocktail Party”.” Neuron 77, no. 5: 980–991. 10.1016/j.neuron.2012.12.037. 23473326 PMC3891478

[ejn70598-bib-0066] Zuk, N. J. , J. W. Murphy , R. B. Reilly , and E. C. Lalor . 2021. “Envelope Reconstruction of Speech and Music Highlights Stronger Tracking of Speech at Low Frequencies.” PLoS Computational Biology 17, no. 9: e1009358. 10.1371/journal.pcbi.1009358. 34534211 PMC8480853

