# Supplementary material for: Enhanced Time‐Locked Decoding for Spoken Words but Not Environmental Sounds in Natural‐Like Auditory Conditions
Source: Eur J Neurosci. 2026 Jul 3;64(1):e70598. doi: 10.1111/ejn.70598 (PMC13329805; doi:10.1111/ejn.70598)
Supplement: Supplementary file 1 — Figure S1: Raw correlation values between the original and reconstructed sounds in the sensor‐level amplitude envelope decoding analysis. The values corresponding to the shortest (0–40 ms) and longest (360–400 ms) latencies are marked, as well as the statistically significant latencies of 120–160 and 160–200 ms. Figure S2: Raw correlation values between the original and reconstructed sounds in the sensor‐level spectrogram decoding analysis. The values corresponding to the shortest (0–40 ms) and longest (360–400 ms) latencies are marked, as well as the statistically significant latencies of 160–200 and 200–240 ms. [file EJN-64-0-s001.pdf]

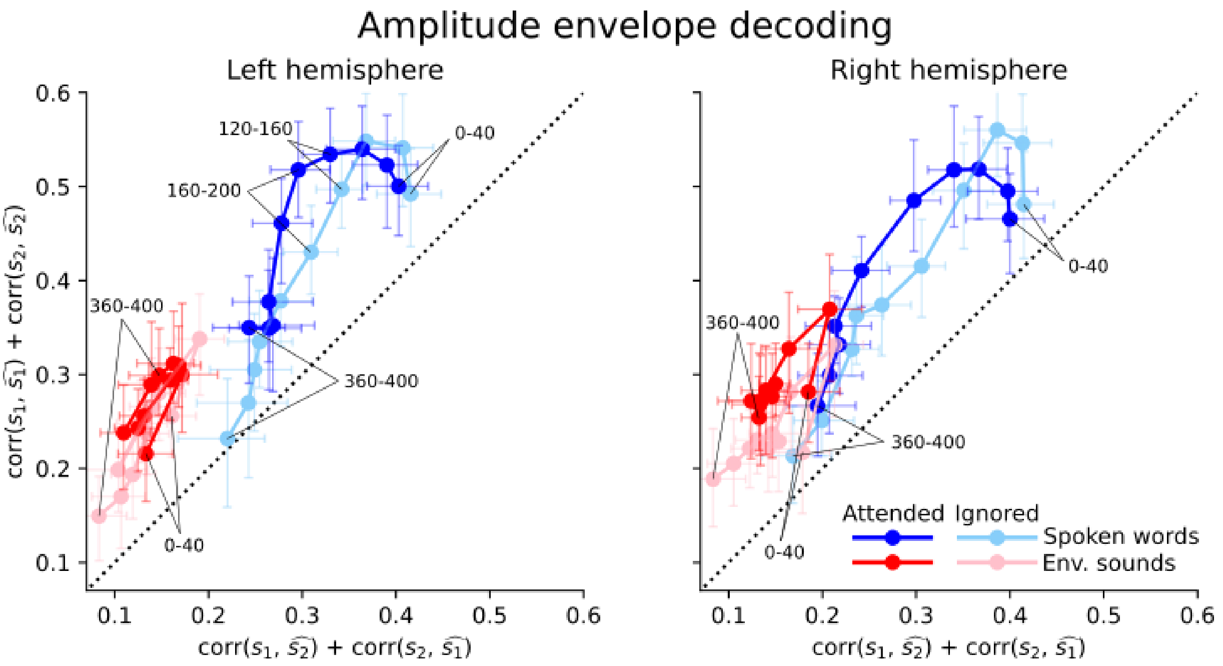

Supplementary Figure 1: Raw correlation values between the original and reconstructed sounds in the sensor-level amplitude envelope decoding analysis. The values corresponding to the shortest (0-40 ms) and longest (360-400 ms) latencies are marked, as well as the statistically significant latencies of 120-160 ms and 160-200 ms.

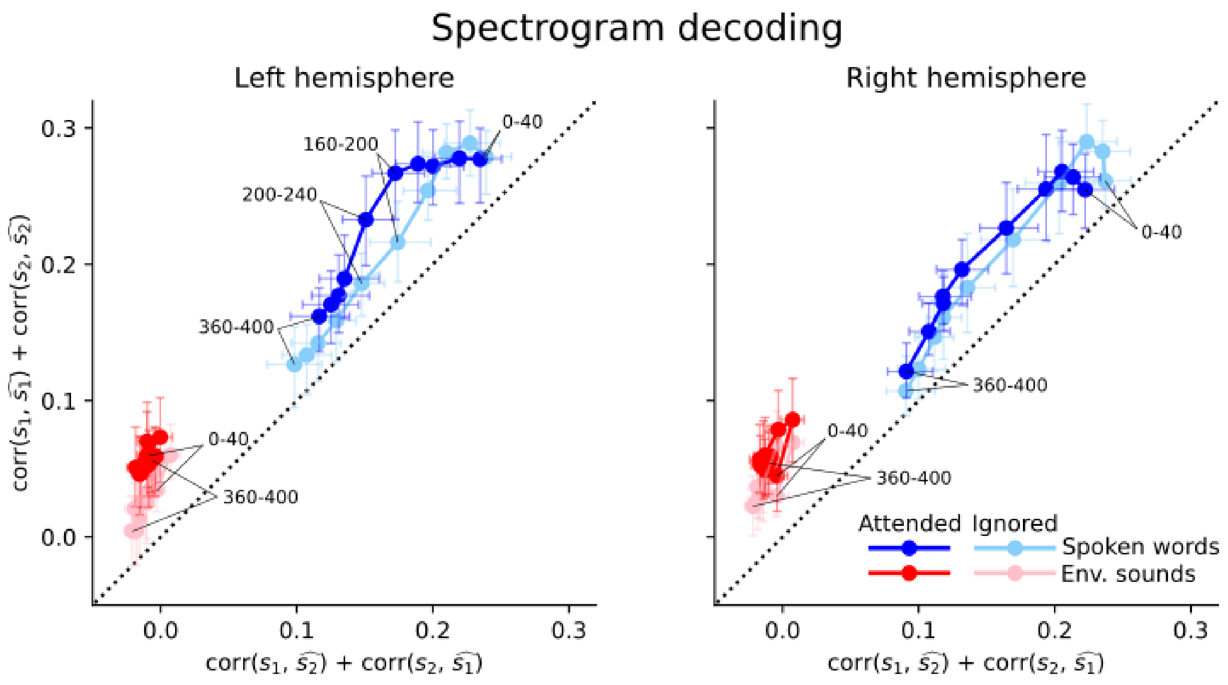

Supplementary Figure 2: Raw correlation values between the original and reconstructed sounds in the sensor-level spectrogram decoding analysis. The values corresponding to the shortest (0-40 ms) and longest (360-400 ms) latencies are marked, as well as the statistically significant latencies of 160-200 ms and 200-240 ms.
